# Personality traits and oral device: a new challenge to increase compliance with MAD therapy for OSAS and snoring

**DOI:** 10.5935/1984-0063.20190124

**Published:** 2020

**Authors:** Luca Mezzofranco, Antonio Luigi Tiberio Gracco, Francesca Milano, Gian Antonio Di-Bernardo, Loris Vezzali, Dino Giovannini

**Affiliations:** 1 University of Padua, Department of Neurosciences - Padova - Italy; 2 Private practice, Private practice - Bologna - Italy; 3 University of Modena and Reggio Emilia, Department of Education and Human Sciences - Reggio Emilia - Italy

**Keywords:** Sleep Apnea Syndromes, Mandibular Advancement, Personality, Anxiety, Dentistry

## Abstract

Obstructive Sleep Apnea Syndrome (OSAS) is a respiratory syndrome and oral devices can be used for its treatment. This study aimed to evaluate the opinions of a generic subject about being treated by a dentist for a general health problem and the association between personality traits and the predisposition to use a MAD for the treatment of OSAS and snorting. One hundred and forty-eight participants were enrolled in the study and were asked to fill in the questionnaires. Personality traits were evaluated using NFC (Need for Closure), PER (openness to new experiences), STAI-Trait and STAI-Stat questionnaires (State-Trait Anxiety Inventory). The propensity to be treated with dental devices for a general health problem such as OSAS and snoring was evaluated with a specific questionnaire. Eight out of ten participants would accept to use dental device to be kept at night for the solution of a health problem or the treatment of a disease that does not affect the teeth. A positive opinion on device used to treat OSAS was associated with higher PER and lower Mad-related distress, while the opinion of usefulness of the device was positively associated with higher PER and STAI-Trait. A positive opinion about treatment of snorting and OSAS using dental devices was associated with higher PER, while lower STAI-Trait was associated with positive opinion on treatment of snorting using dental-devices. The results suggest that some personality traits are associated with the propensity to use MAD to treat a general pathology as OSAS.

## INTRODUCTION

Obstructive Sleep Apnea Syndrome (OSAS) is a respiratory syndrome caused by repetitive collapse of the upper airway^1^. Clinical definition includes loud snoring, witnessed breathing interruptions, daytime sleepiness and awakenings due to gasping or choking in the presence of at least five obstructive respiratory events (i.e. apneas or hypopneas) per hour of sleep^1^. OSAS is common disorder in adult population^1-3^ and may cause impaired mental concentration, decreased alertness, tiredness or fatigue, headaches, and irascible temper^4^. In addition, OSAS may contribute to the deterioration of patients with cardiovascular or pulmonary disease^4,5^.

Therapeutic options for OSAS include behavioral therapy (i.e. diet and exercise, weight loss, positional therapy, and oropharyngeal exercise) and specific therapies such as Continuous Positive Airway Pressure (CPAP), roncosurgery or maxillofacial surgery^1,6^. CPAP is considered the gold standard treatment^7^ but the effectiveness is likely to depend on adequate compliance. Adherence to CPAP is considered good if the device is used for more than 4 hours/night for at least 70% of the nights^8^, but actual long-term adherence lies between 46% and 80%^9-11^.

**Figure 1:**
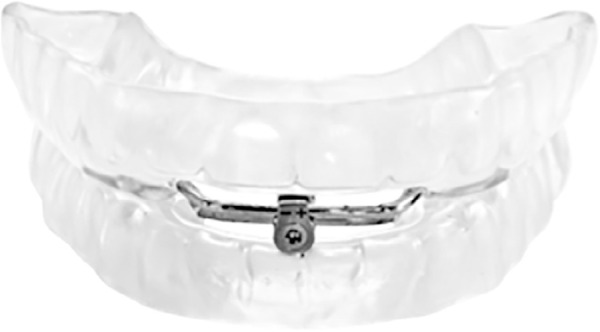
Mandibular Advancement Device

Recent studies suggest that treatment of OSAS may involve the dentist in a multidisciplinary approach including the use of oral appliances such as Mandibular Advancement Devices (MADs) (Figure 1)^12^. MADs are oral appliances that hold the lower jaw and tongue forward thus creating more room to breathe and preventing snoring^13^. Although not achieving the efficacy of CPAP in decreasing nocturnal respiratory events, MAD may provide similar benefits with respect to CPAP^11,14^. The high compliance of MADs can play a significant role in achieving these results^14^. Previous studies estimated MADs compliance of 77% at 1 year and 65% at 10 years, with MAD use for at least 6 nights/week in 55% of the patients^9^.

Since the potential benefits of MADs rely on the high compliance, identifying the subjects who are willing to using MAD is crucial to achieve the expected results. This study aimed to evaluate the opinions of a generic subject about being treated by a dentist for a general health problem and the association between personality traits and the predisposition to use a MAD for the treatment of OSAS.

## METHODS

### Study design

This is a pilot study evaluating the resistance of generic subjects to be treated by a dentist for a general health problem and the association between personality traits and the predisposition to use a MAD for the treatment of OSAS. The evaluation was based on anonymized questionnaires, which were available at the waiting room in three dental wards in Padua, Ferrara and Bologna (Italy). Subjects referring to the three dental wards were free to fill the anonymized questionnaires, which did not include any personal data or identifier. The filled forms were collected in an opaque paper box placed in the waiting room in the dental wards.

### Questionnaires

Each study form included the Italian short version of the need for closure scale (NFC)^15^, the 10 items regarding the domain “openness to new experiences” of the Big Five Inventory^16^ and the Spielberger STAI-Y (State-Trait Anxiety Inventory)^17^.

The Italian short version of the NFC consisted of 14 items rated on 6-point Likert scales with higher scores reflecting greater need for closure^15^. Need for closure is defined as “an aversion toward ambiguity and the desire for firm answers - any answers, as long as they allow one to avoid confusion and uncertainty”^18^.

The Big Five Inventory is a self-report inventory designed to measure the Big Five dimensions (openness to experience, conscientiousness, extraversion, agreeableness, and neuroticism)^16^. In this study, we evaluated the domain “openness to new experiences” (OPEN) which measures the degree of intellectual curiosity, creativity and preference for novelty. The domain is based on 10 items rated as 5-point Likert scales and higher scores are correlated with more openness to new experiences.

The STAI-Y is based on 20 items evaluating state anxiety (STAI-State) and 20 items evaluating trait anxiety (STAI-Trait). Items are rated as 4-point Likert scales and higher scores are correlated with higher levels of anxiety^17^. In this study, STAI-Y scores were calculated as mean rather than sum of single items due to some incomplete items.

In addition, each study form has also a section that was specifically built for the study aim and included 15 items about dental care and dental devices, a brief presentation of OSAS, snoring and MADs (with a picture), and 15 items about MADs. Two items (regarding concerns about having MAD limiting mandibular movements during nighttime and worries about keeping a device in the mouth during nighttime) were summarized in a single score (MAD-related worries, MAD-W) with higher score associated with more concerns about using MAD during nighttime.

Additional data (age, sex, university student vs. worker) were also provided by each participant in the anonymized form.

### Statistical analysis

Continuous data were expressed as median and interquartile range (IQR), and categorical data as number and percentage. Continuous data were compared among groups using Mann-Whitney test or Kruskal-Wallis test, as appropriate. Correlation between continuous data was assessed using Spearman’s rank correlation coefficient. Categorical data were compared among groups using Fisher’s exact test. All test were 2-sided and a *p*-value less than 0.05 was considered statistically significant. Statistical analysis was performed using R 3.2.2 software (R Foundation for Statistical Computing, Vienna, Austria)^19^.

## RESULTS

### Participants

One hundred and forty-eight participants (35 males and 113 females) filled the questionnaires and were included in the study. Median age was 30 years (IQR 21-45). There were 65 university students (45%) and 82 workers (55%).

### Scores

**Figure 2:**
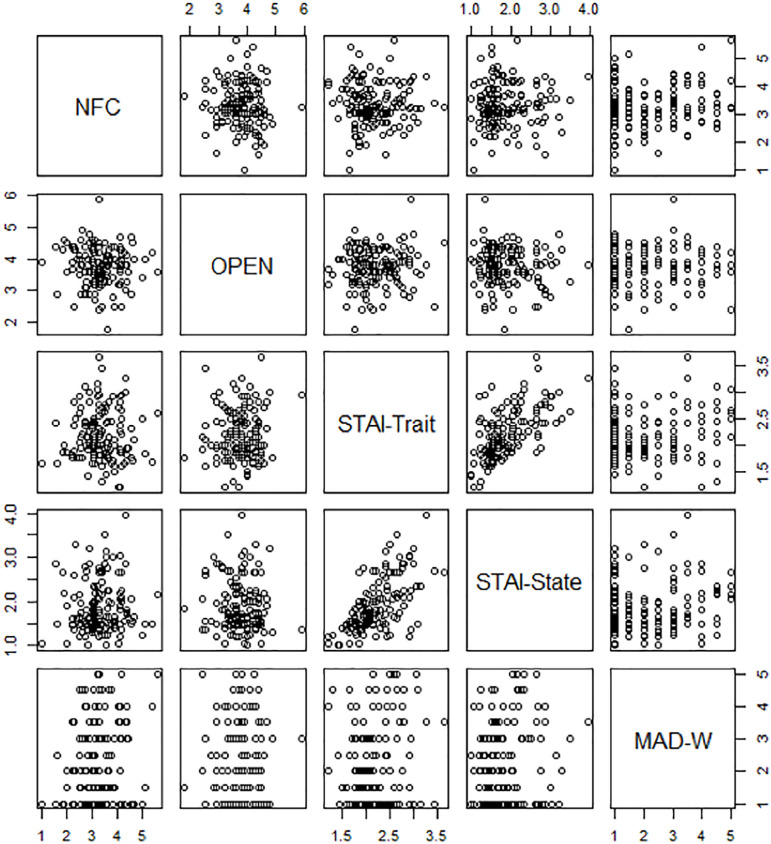
Correlation between scores: scatterplot.

Median scores (with IQR) are shown in Table 1. STAI-Trait and STAI-State were correlated (Spearman rho coefficient 0.63, *p*<0.0001), while no significant correlations were observed between any other pair of scores (Figure 2 and Supplementary Table 1).

**Table 1:** Scores.

	Median (IQR)
NFC	3.3 (IQR 3.0-3.8)
OPEN	3.8 (3.5-4.2)
STAI-Trait	2.1 (1.8-2.5)
STAI-State	2 (1-3)
MAD-W ^a^	1.7 (1.5-2.2)

a Not available in five participants.

**Supplementary Table 1:** Correlation between scores

	NFC	PER	Trait-STAI	State-STAI	ansiaMAD
NFC	-	-0.08 (*p*=0.32)	-0.03 (*p*=0.71)	0.09 (*p*=0.28)	0.04 (*p*=0.62)
ANS	-	-	-0.01 (*p*=0.94)	-0.09 (*p*=0.29)	-0.01 (*p*=0.87)
Trait-STAI	-	-	-	0.63 (*p*<0.0001)	0.15 (*p*=0.07)
State-STAI	-	-	-	-	0.13 (*p*=0.13)
ansiaMAD	-	-	-	-	-

Data expressed as Spearman correlation coefficient with *p*-value in brackets.

### Association between scores and participant characteristics

The association between scores and participant characteristics is shown in Table 2. Higher NFC was associated with older age (*p*<0.0001) and worker group (*p*<0.0001), while higher STAI-Trait was associated with younger age (*p*=0.0007) and student group (*p*=0.0003). OPEN, STAI-State and MAD-W were not associated with age or student/worker group (Table 2). Males and females showed similar scores (Table 2).

**Table 2:** Scores and participant characteristics.

	N	NFC	OPEN	STAI-Trait	STAI-State	MAD-W
Age a	148	0.43	-0.02	-0.28	-0.12	0.00
		(*p*<0.0001)	(*p*=0.82)	(*p*=0.0007)	(*p*=0.17)	(*p*=0.99)
Sex:						
Male	35	3.3 (2.9-3.9)	3.8 (3.5-4.2)	2.1 (1.9-2.5)	1.7 (1.5-2.3)	2.0 (1.0-3.0)
Female	113	3.1 (2.8-3.5)	3.7 (3.4-4.3)	2.1 (1.8-2.3)	1.8 (1.5-2.1)	2.0 (1.5-3.0)
		(*p*=0.09)	(*p*=0.81)	(*p*=0.56)	(*p*=0.88)	(*p*=0.77)
Subject:						
Workers	82	3.5 (3.1-4.1)	3.8 (3.5-4.1)	2.0 (1.8-2.4)	1.7 (1.5-2.1)	2.0 (1.0-3.0)
Students	66	3.1 (2.7-3.3)	3.8 (3.3-4.3)	2.3 (2.0-2.7)	1.7 (1.5-2.4)	2.0 (1.0-3.0)
		(*p*<0.0001)	(*p*=0.53)	(*p*=0.0003)	(*p*=0.17)	(*p*=0.82)

Data expressed as median (IQR) or ^a^Spearman correlation coefficient.

### Association between scores and opinions about dental care and dental devices

The majority of the participants reported previous experience in receiving dental care, but such experience was not associated with any scores (items 1 to 5; Table 3). A favorable opinion on dental alignment devices was associated with a lower MAD-W (item 6: Spearman’s rho -0.20, *p*=0.02). The majority of participants (80%) would accept to use a dental device during nighttime for non-dental disease, and they had lower STAI-Trait than those who would not (item 9: median 2.0 *vs.* 2.2, *p*=0.03; Table 3). Only five participants had already been visited or treated for OSAS. Most participants (60%) already knew about OSAS, and they had higher OPEN than those who did not (item 11: median 3.9 *vs.* 3.6, *p*=0.0009; Table 3). One third of participants already received information about OSAS by clinicians, and they had higher NFC than those who did not (item 12: median 3.5 *vs.* 3.2, *p*=0.03; Table 3). A higher NFC was associated with snorting partners but not with snorting participants (items 14 and 15; Table 3).

**Table 3:** Scores and opinions about dental care and dental devices.

#	Item	N	NFC	OPEN	STAI-Trait	STAI-State	MAD-W
**1**	**Previous dental care b**						
	No	16	3.3 (2.9-3.7)	3.9 (3.6-4.4)	2.1 (2.0-2.5)	1.8 (1.5-2.4)	2.5 (1.3-3.3)
	Yes	129	3.3 (2.9-3.8)	3.8 (3.4-4.1)	2.1 (1.8-2.5)	1.7 (1.5-2.2)	2.0 (1.0-3.0)
			(*p*=0.83)	(*p*=0.30)	(*p*=0.59)	(*p*=0.54)	(*p*=0.62)
**2**	**Knowledge about dental device c**						
	No	8	3.3 (2.9-3.8)	3.6 (3.2-4.0)	2.2 (1.9-2.5)	1.8 (1.5-2.3)	2.0 (1.4-2.5)
	Yes	138	3.3 (2.9-3.8)	3.8 (3.5-4.2)	2.1 (1.8-2.5)	1.7 (1.5-2.2)	2.0 (1.0-3.0)
			(*p*=0.95)	(*p*=0.21)	(*p*=0.57)	(*p*=0.89)	(*p*=0.97)
**3**	**Mental association with the word "dental device" de**						
	Positive	31	3.2 (2.9-3.8)	3.8 (3.7-4.3)	2.2 (1.8-2.5)	1.7 (1.5-2.1)	1.5 (1.03-2.6)
	Neutral	67	3.3 (2.9-3.6)	3.8 (3.4-4.2)	2.1 (1.9-2.5)	1.7 (1.5-2.3)	2.0 (1.0-3.0)
	Negative	35	3.3 (3.0-3.7)	3.8 (3.6-4.2)	2.1 (1.8-2.7)	1.7 (1.5-2.2)	2.5 (1.3-3.8)
			(*p*=0.71)	(*p*=0.52)	(*p*=0.83)	(*p*=0.95)	(*p*=0.11)
**4**	**Previous sight about dental device b**						
	No	11	3.3 (2.8-3.9)	3.7 (3.4-4.3)	1.9 (1.8-2.1)	1.4 (1.3-1.8)	2.0 (1.8-3.0)
	Yes	134	3.3 (2.9-3.8)	3.8 (3.5-4.2)	2.1 (1.9-2.5)	1.7 (1.5-2.2)	2.0 (1.0-3.0)
			(*p*=0.70)	(*p*=0.62)	(*p*=0.08)	(*p*=0.08)	(*p*=0.64)
**5**	**Scenario of previous sight about dental device f**						
	Dentist ward	15	3.1 (3.0-3.5)	3.8 (3.2-4.0)	2.2 (2.0-2.6)	2.1 (1.5-2.8)	2.0 (1.6-2.9)
	Own experience	66	3.3 (2.9-3.9)	3.8 (3.5-4.2)	2.1 (1.8-2.5)	1.7 (1.5-2.2)	1.5 (1.0-3.0)
	Relatives	23	3.6 (3.1-3.7)	3.8 (3.6-4.0)	2.0 (1.8-2.4)	1.7 (1.5-2.0)	2.5 (1.1-3.5)
	Friends	19	3.4 (2.9-3.9)	3.8 (3.6-4.2)	2.2 (1.8-2.8) (*p*=0.61)	1.6 (1.5-2.1)	2.5 (1.5-4.0)
			(*p*=0.56)	(*p*=0.54)		(*p*=0.37)	(*p*=0.12)
**6**	**Personal opinion on dental alignment devices (from 1 negative to- 10 positive) ag**						
		143	0.10	0.09	-0.08	-0.11	-0.20
			(*p*=0.25)	(*p*=0.27)	(*p*=0.32)	(*p*=0.19)	(*p*=0.02)
**7**	**Personal opinion on possibility of using dental devices for non-dental diseases g**						
	No	53	3.1 (2.8-3.6)	3.8 (3.3-4.1)	2.2 (1.9-2.5)	1.7 (1.5-2.1)	2.0 (1.5-3.0)
	Yes	90	3.4 (3.1-4.1)	3.9 (3.5-4.3)	2.0 (1.9-2.4) (*p*=0.23)	1.7 (1.5-2.2)	2.0 (1.0-3.0)
			(*p*=0.06)	(*p*=0.29)		(*p*=0.79)	(*p*=0.33)
**8**	**Reason for negative answer above g**						
	No idea about alternative uses	25	3.1 (2.7-3.3)	3.7 (3.3-4.0)	2.2 (1.8-2.4)	1.6 (1.5-2.2)	2.5 (1.5-4.0)
	Useless for non-dental diseases	18	3.0 (2.7-3.7)	4.0 (3.2-4.3)	2.1 (1.9-2.4)	1.7 (1.5-2.1)	2.0 (1.1-2.0)
	Useless pain	5	3.3 (2.9-3.6)	3.8 (3.6-4.1)	2.3 (2.1-2.8)	1.7 (1.6-1.7)	3.0 (1.5-3.0)
			(*p*=0.73)	(*p*=0.45)	(*p*=0.35)	(*p*=0.86)	(*p*=0.19)
**9**	**Propensity of using of dental device during nighttime for non-dental disease hi**						
	No	25	3.1 (2.8-3.4)	3.6 (3.3-4.0)	2.2 (2.1-2.7)	1.7 (1.5-2.5)	2.0 (1.5-3.0)
	Yes	119	3.3 (3.0-3.9)	3.8 (3.5-4.3)	2.0 (1.8-2.5)	1.7 (1.5-2.1)	2.0 (1.0-3.0)
			(*p*=0.08)	(*p*=0.09)	(*p*=0.03)	(*p*=0.26)	(*p*=0.46)
**11**	**Previous knowledge about OSAS c**						
	No	55	3.2 (2.9-3.7)	3.6 (3.3-4.0)	2.1 (1.9-2.5)	1.7 (1.5-2.2)	2.0 (1.0-3.0)
	Yes	91	3.6 (3.0-3.9)	3.9 (3.6-4.3)	2.1 (1.8-2.4)	1.7 (1.5-2.2)	2.0 (1.0-3.0)
			(*p*=0.36)	(*p*=0.0009)	(*p*=0.92)	(*p*=0.88)	(*p*=0.65)
**12**	**Dentist/clinician provided information on harms due to OSAS c**						
	No	97	3.2 (2.9-3.6)	3.8 (3.4-4.2)	2.1 (1.9-2.5)	1.7 (1.5-2.2)	2.0 (1.1-3.0)
	Yes	49	3.5 (3.1-4.2)	3.9 (3.6-4.2)	2.1 (1.8-2.4)	1.8 (1.5-2.2)	2.0 (1.0-3.0)
			(*p*=0.03)	(*p*=0.34)	(*p*=0.69)	(*p*=0.17)	(*p*=0.44)
**14**	**Snoring during sleep b**						
	Never	52	3.1 (2.8-3.7)	3.8 (3.3-4.2)	2.0 (1.8-2.3)	1.7 (1.5-2.1)	2.0 (1.1-2.9)
	Sometimes	76	3.4 (3.1-3.9)	3.8 (3.4-4.2)	2.2 (1.8-2.6)	1.8 (1.5-2.2)	2.0 (1.0-3.0)
	Often/always	17	3.3 (2.9-3.7)	3.8 (3.6-4.3)	2.1 (1.9-2.4)	1.7 (1.4-2.1)	2.0 (1.0-3.0)
			(*p*=0.17)	(*p*=0.61)	(*p*=0.60)	(*p*=0.57)	(*p*=0.87)
**15**	**Snoring sleeping partner b**						
	Never	45	3.1 (2.9-3.8)	3.9 (3.6-4.3)	2.0 (1.8-2.2)	1.6 (1.5-2.0)	1.5 (1.0-3.0)
	Sometimes	44	3.2 (2.8-3.9)	3.6 (3.2-4.0)	2.2 (1.8-2.7)	1.8 (1.5-2.2)	2.5 (1.5-3.3)
	Often	42	3.3 (3.0-3.5)	3.8 (3.6-4.2)	2.4 (2.0-2.5)	1.8 (1.5-2.7)	2.0 (1.1-3.0)
	Always	15	3.8 (3.5-4.5)	3.9 (3.6-4.0)	2.1 (1.9-2.4)	1.7 (1.4-2.1)	1.0 (1.0-3.0)
			(*p*=0.004)	(*p*=0.14)	(*p*=0.07)	(*p*=0.29)	(*p*=0.55)

Data expressed as median (IQR) or a Spearman correlation coefficient. Data not available in b3, c2, d15, f11, g5 and h4 participants. ^e^Positive associations included 42 "improvements"; neutral associations included 42 "dentistry devices", 8 "childhood and past experience", 11 "dentist" and 6 "other"; negative associations included 30 "pain, discomfort, fear" and 5 "unaesthetic ". ^i^Three participants referred to possible sleeping problems, 10 stated that "it is useful only for dental problems" and 9 believed it could be useless or annoying, while 3 participants did not answer.

### Association between scores and opinions about MAD

A favorable opinion on MAD and its benefits was associated with higher OPEN and lower MAD-W (items 16 and 18, Table 4). According to the participants, the main advantages of MAD could be better sleeping and breathing quality (58 participants), prevention of general health problems (43 participants) and comfort (18 participants). On the other hand, the main disadvantages could be discomfort/pain (62 participants), cost (14 participants) and esthetic issues (5 participants). The remaining participants did not report any specific opinion on advantages or disadvantages.

**Table 4:** Scores and opinions about MAD.

#	Item	N	NFC	OPEN	STAI-Trait	STAI-State	MAD-W
**16**	**Opinion about MAD (from 1 negative to- 10 positive) ^ab^**	141	0.01	0.20	-0.08	0.02	-0.19
			(*p*=0.97)	(*p*=0.02)	(*p*=0.35)	(*p*=0.78)	(*p*=0.03)
**18**	**Opinion about benefits of MAD (from 1 negative to- 10 positive) (1-10) ^ab^**	141	0.01	0.21	0.07	0.03	-0.20
			(*p*=0.89)	(*p*=0.01)	(*p*=0.44)	(*p*=0.74)	(*p*=0.01)
**22**	**Usefulness of MAD (from 1 useless to 5 useful)^ c^**	4 (4-5)	-0.06	0.18	0.18 (*p*=0.04)	0.06	-0.14
			(*p*=0.50)	(*p*=0.03)		(*p*=0.50)	(*p*=0.11)
**23**	**MAD is not needed when sleeping alone (from 1 disagree to 5 agree) ^d^**	1 (1-2)	0.08	-0.21 (*p*=0.01)	0.05	0.08	0.22
			(*p*=0.35)		(*p*=0.54)	(*p*=0.32)	(*p*=0.008)
**24**	**Snoring people should use a device to avoid annoying sleeping partners (from 1 disagree to 5 agree) ^d^**	4 (3-4)	0.13	0.14	-0.06	-0.12	0.01
			(*p*=0.13)	(*p*=0.10)	(*p*=0.46)	(*p*=0.14)	(*p*=0.95)
**25**	**Dental devices are not useful to prevent stroke or heart attack (from 1 disagree to 5 agree) ^d^**	2 (1-3)	0.12	-0.20 (*p*=0.02)	-0.06	-0.07	0.21
			(*p*=0.15)		(*p*=0.44)	(*p*=0.42)	(*p*=0.01)
**26**	**Worries about keeping a device in the mouth during nighttime (from 1 disagree to 5 agree) ^d^**	2 (1-3)	0.05	-0.02	0.12	0.12	This item is *p*art of the score.
			(*p*=0.58)	(*p*=0.86)	(*p*=0.14)	(*p*=0.15)	
**27**	**Distrust in dental devices (from 1 disagree to 5 agree) ^d^**	1 (1-2)	0.19	-0.15	0.05	0.18 (*p*=0.03)	0.48
			(*p*=0.03)	(*p*=0.07)	(*p*=0.53)		(*p*<0.0001)
**28**	**Concerns about having MAD limiting mandibular movements during nighttime (from 1 disagree to 5 agree) ^d^**	2 (1-3)	0.04	-0.01	0.16	0.14	This item is *p*art of the score.
			(*p*=0.61)	(*p*=0.90)	(*p*=0.06)	(*p*=0.11)	
**29-a**	**Opinion about dental devices for treatment of snoring (from 1 negative to 10 positive)^ e^**	8 (7-10)	0.14	0.28 (*p*=0.0009)	-0.18 (*p*=0.04)	-0.11	-0.13
			(*p*=0.10)			(*p*=0.22)	(*p*=0.12)
**29-b**	**Opinion about dental devices for treatment of OSAS (from 1 negative to 10 positive) ^e^**	9 (8-10)	0.04	0.20	0.03	-0.02	-0.13
			(*p*=0.68)	(*p*=0.02)	(*p*=0.71)	(*p*=0.78)	(*p*=0.12)

Data expressed as median (IQR) or ^a^ Spearman correlation coefficient. Data not available in ^b^7, ^c^9, ^d^5 and ^e^11 participants.

The opinion on the usefulness of MAD was positively associated with higher OPEN and STAI-Trait (item 22, Table 4).

Participants that agreed about MAD not needed when sleeping alone had also lower OPEN and higher MAD-W (item 23, Table 4). Participants that agreed about dental devices not useful to prevent stroke or heart attack had also lower OPEN and higher MAD-W (item 25, Table 4). Greater distrust on dental devices was associated with higher NFC, STAI-State and MAD-W (item 27, Table 4). A positive opinion about treatment of snorting and OSAS using dental devices was associated with higher OPEN, while lower STAI-Trait was associated with positive opinion on treatment of snorting using dental devices (items 29a and 29b, Table 4).

## DISCUSSION

This study aimed to evaluate personal opinions and personality traits regarding the predisposition to use a MAD for the treatment of OSAS. The majority of participants would accept to use a dental device during nighttime for non-dental disease. A favorable opinion on dental devices (including dental alignment devices and MADs) and knowledge of OSAS were associated with less concern about using MAD during nighttime.

In treatment of OSAS, MAD may provide similar benefits with respect to CPAP, thanks to high compliance of MAD^14^. Therefore, identifying the subjects who are willing to using MAD is crucial to achieve the expected results. Our data suggested that a favorable opinion on dental alignment devices was associated with a less concern about using MAD during nighttime, while distrust on dental devices was associated with more concern about using MADs.

This finding might suggest an important role of previous experience with other dental devices. Moreover, openness to new experiences was associated with a favorable opinion on MAD and on its usefulness. Subjects with higher degree of intellectual curiosity and preference for novelty may better accept the use of a dental device for the care of non-dental disease, thus leading to increased compliance in MAD use.

In our study, 80% of participants would accept to use a dental device during nighttime for non-dental disease. This finding was in agreement with previous studies reporting a high acceptability of using a dental device for non-dental disease. Participants indicated better sleeping/breathing quality and prevention of general health problems as the main advantages of MAD, thus suggesting a good comprehension of the primary aim of the device.

It is noteworthy that discomfort/pain was indicated as the main disadvantage of MAD, while the cost of the device was not considered a strong issue. Communication between dentist and patient should focus on primary advantages of MAD regarding sleeping/breathing quality and general health, and on reassuring the patient about limited discomfort/pain. A previous study reported a good management of discomfort/pain thanks to mandibular exercises^20^.

In our study, a limited proportion of participants already knew about OSAS and received information about harms due to OSAS by clinicians. In addition, participants with more concern about using MADs believed that it was not needed when sleeping alone. Interestingly, knowledge about OSAS was associated with increased openness to new experiences, thus an adequate introduction of OSAS before suggesting the use of MAD may improve patient’s awareness and acceptability of MAD. Some participants underestimated the benefit of MAD regarding prevention of stroke or heart attack. Introduction of OSAS should appropriately address possible harms associated with OSAS while taking into account that receiving information about harms due to OSAS was associated with increased need for closure in our participants.

The dentist may play an important role in the screening for OSAS, because dental care of most people includes an annual dental visit. In 2014, the Italian Guidelines on Dental Prevention and Treatment of OSAS stressed the importance of the dentist in the screening for OSAS and suggested that dentists should assess the presence of OSAS during patient interview^21^. In addition, the dentist is responsible for the management of oral appliances and patient compliance. Therefore, appropriate education of both professionals and dental students on OSAS should be warranted^21^.

This is the first study that evaluated the opinions about using MAD for OSAS treatment among general subjects who are not receiving treatment for OSAS. However, the study has also some limitations. First, the generalizability of the findings is limited because only Italian participants were included. Second, MAD-W score was not a validated instrument but was built for the purpose. In addition, MAD-W was not correlated with need for closure, anxiety or openness to new experiences, but it was unclear whether this result might be due to low precision of the two items summarized in the MAD-W score.

## CONCLUSIONS

The study found that most patients (80%) would accept to be treated by a dentist with an oral device for a general health problem. A more positive opinion was found in people with a lower STAI-State and in those with previous positive orthodontic experiences, while a more negative opinion was associated with a higher degree of NFCand a higher level of anxiety.

The younger ones and the students showed a higher STAI-Trait, while a more advanced age and worker status were associated with a greater NFC.

To improve the generalizability of the sample it is desirable in the future to involve other categories of patients as students in other educational institutions or unemployed or retired.

Knowing how to investigate specific psychological resistance should be part of the expertise of the expert in sleep disorders, as knowing patient personality traits can play a key role in selecting and managing compliance with MAD. To understand more in depth the personalized approach to MAD therapy it will also be useful to study further psychological traits.

We also reconsidered how the population needs to be more aware of the syndrome of obstructive sleep apnea, as only 34% of the sample said they received information on OSAS from a doctor, and a knowledge of the disease found in 62%.

## References

[1] Epstein LJ, Kristo D, Strollo PJ Jr, Friedman N, Malhotra A, Patil SP, Adult Obstructive Sleep Apnea Task Force of the American Academy of Sleep Medicine (2009). Clinical guideline for the evaluation, management and long-term care of obstructive sleep apnea in adults. J Clin Sleep Med.

[2] Heinzer R, Vat S, Marques-Vidal P, Marti-Soler H, Andries D, Tobback N (2015). Prevalence of sleep-disordered breathing in the general population: THE HypnoLaus study. Lancet Respir Med.

[3] Gracco A, Bruno G, de Stefani A, Ragona RM, Mazzoleni S, Stellini E (2017). Combined Orthodontic and Surgical Treatment in a 8-Years-Old Patient Affected By Severe Obstructive Sleep Apnea: A Case-Report. J Clin Pediatr Dent.

[4] Whitelaw WA, Burgess KR (2010). Diagnosis of sleep apnoea: Some critical issues. Indian J Med Res.

[5] Rudnicka A, Plywaczewski R, Jonczak L, Górecka D, Sliwinski P (2010). Prevalence of stroke in patients with obstructive sleep apnoea. Pneumonol Alergol Pol.

[6] Sundaram S, Lim J, Lasserson TJ (2005). Surgery for obstructive sleep apnoea in adults. Cochrane Database Syst Rev.

[7] Spicuzza L, Caruso D, Di Maria G (2015). Obstructive sleep apnoea syndrome and its management. Ther Adv Chronic Dis.

[8] Schwab RJ, Badr SM, Epstein LJ, Gay PC, Gozal D, Kohler M, ATS Subcommittee on CPAP Adherence Tracking Systems (2013). An official American Thoracic Society statement: continuous positive airway pressure adherence tracking systems. The optimal monitoring strategies and outcome measures in adults. Am J Respir Crit Care Med.

[9] Basyuni S, Barabas M, Quinnell T (2018). An update on mandibular advancement devices for the treatment of obstructive sleep apnoea hypopnoea syndrome. J Thorac Dis.

[10] Phillips CL, Grunstein RR, Darendeliler MA, Mihailidou AS, Srinivasan VK, Yee BJ (2013). Health outcomes of continuous positive airway pressure versus oral appliance treatment for obstructive sleep apnea: a randomized controlled trial. Am J Respir Crit Care Med.

[11] Doff MH, Hoekema A, Wijkstra PJ, van der Hoeven JH, Huddleston Slater JJ, de Bont LG (2013). Oral appliance versus continuous positive airway pressure in obstructive sleep apnea syndrome: a 2-year follow-up. Sleep.

[12] Lim J, Lasserson TJ, Fleetham J, Wright J (2004). Oral appliances for obstructive sleep apnoea. Cochrane Database Syst Rev.

[13] Mintz SS, Kovacs R (2018). The use of oral appliances in obstructive sleep apnea: a retrospective cohort study spanning 14 years of private practice experience. Sleep Breath.

[14] Ramar K, Dort LC, Katz SG, Lettieri CJ, Harrod CG, Thomas SM (2015). Clinical Practice Guideline for the Treatment of Obstructive Sleep Apnea and Snoring with Oral Appliance Therapy: An Update for 2015. J Clin Sleep Med.

[15] Pierro A, Sheveland A, Livi S, Kruglanski AW (2015). Person-Group Fit on the Need for Cognitive Closure as a Predictor of Job Performance, and the Mediating Role of Group Identification. Group Dyn.

[16] Fossati A, Borroni S, Marchione D, Maffei C (2011). The Big Five Inventory (BFI): Reliability and validity of its Italian translation in three independent nonclinical samples. Eur J Psychol Assess.

[17] Barnes LLB, Harp D, Jung WS (2002). Reliability generalization of scores on the spielberger state-trait anxiety inventory. Educ Psychol Meas.

[18] Kruglanski AW, Sorrentino RM, Higgins T (1990). Motivations for Judging and Knowing: Implications for causal Attribution. Handbook of Motivation and Cognition, Volume 1: Foundations of Social Behavior.

[19] Team RC (2016). R: A language and environment for statistical computing.

[20] Cunali PA, Almeida FR, Santos CD, Valdrichi NY, Nascimento LS, Dal-Fabbro C (2011). Mandibular exercises improve mandibular advancement device therapy for obstructive sleep apnea. Sleep Breath.

[21] Polimeni A, Attanasi P, Barbato E, Cappello G, Cozza PD, Delogu P (2014). Linee guida nazionali per la prevenzione ed il trattamento odontoiatrico della Sindrome delle Apnee Ostruttive nel Sonno (OSAS).

